# Adaptive Data Synchronization Algorithm for IoT-Oriented Low-Power Wide-Area Networks

**DOI:** 10.3390/s18114053

**Published:** 2018-11-20

**Authors:** Andrea Petroni, Francesca Cuomo, Leonisio Schepis, Mauro Biagi, Marco Listanti, Gaetano Scarano

**Affiliations:** Department of Information, Electrical and Telecommunication Engineering (DIET), “Sapienza” University of Rome, 00184 Rome, Italy; francesca.cuomo@uniroma1.it (F.C.); schepis.1533794@studenti.uniroma1.it (L.S.); mauro.biagi@uniroma1.it (M.B.); marco.listanti@uniroma1.it (M.L.); gaetano.scarano@uniroma1.it (G.S.)

**Keywords:** Internet of Things, LPWANs, rdiff, synchronization

## Abstract

The Internet of Things (IoT) is by now very close to be realized, leading the world towards a new technological era where people’s lives and habits will be definitively revolutionized. Furthermore, the incoming 5G technology promises significant enhancements concerning the Quality of Service (QoS) in mobile communications. Having billions of devices simultaneously connected has opened new challenges about network management and data exchange rules that need to be tailored to the characteristics of the considered scenario. A large part of the IoT market is pointing to Low-Power Wide-Area Networks (LPWANs) representing the infrastructure for several applications having energy saving as a mandatory goal besides other aspects of QoS. In this context, we propose a low-power IoT-oriented file synchronization protocol that, by dynamically optimizing the amount of data to be transferred, limits the device level of interaction within the network, therefore extending the battery life. This protocol can be adopted with different Layer 2 technologies and provides energy savings at the IoT device level that can be exploited by different applications.

## 1. Introduction

The Internet of Things has now become reality and is expected to have by the 2020s over a billion devices connected to the Internet, everywhere, at any time [1]. Smart homes, wearable devices, smart cities, health care, transportation, and farming represent just few reference scenarios where the application of IoT-based models would be successful. The goal of IoT is to realize an environment within which *things* are uniquely identified and able to interact with one another through the exchange of information. Moreover, the development of this new paradigm has been sped up thanks to incoming 5G technology that will provide ultimate performance in terms of data rates, latency, and network coverage. Therefore, the concept of connectivity among devices will soon be completely revolutionized.

Providing good performance is paramount when dealing with mobile communications and, in general, with real-time services that request high data rates and low latency. However, there are many other application scenarios where the sporadic interaction among devices makes energy saving the main aspect to take care of. In this regard, Bluetooth Low Energy, ZigBee, and Low-Power Wi-Fi will be used in a large part of the so-called *consumer* IoT (cIoT) market [2] that refers to all those applications aiming to improve citizen life quality. Specifically, the technologies cited above are tailored to Personal Area Networks (PANs) as they essentially provide single-user coverage area. The Internet Engineering Task Force (IETF) has recently standardized several IoT-oriented protocols, such as the one considering the use of IPv6 over Low-Power Wireless Personal Area Networks (identified as 6LoWPAN) [3,4]. Several devices especially employed for health and fitness activity monitoring have been already implemented with the 6LoWPAN protocol suite and launched into the market.

In parallel to the cIoT, the world of *industrial* IoT (iIoT) has been recently rising. The iIoT collects those scenarios where information technologies are integrated into industrial and public facilities contexts, to make activity management and monitoring more efficient [5]. As with cIoT, many iIoT applications are characterized by limited device mobility and sporadic data transmission, but on the other hand a wider coverage area than in PAN-based services is requested. The satisfaction of these requirements has been achieved through the development of specific radio access technologies, referred to as Low-Power Wide-Area Networks (LPWANs), that best match the features of iIoT. Several standards and vendors in the field of LPWANs have emerged during the last decade [6]. The first one was Sigfox [7] that in 2009 presented on the market its ultra-narrow band (UNB) patented technology. A few years later, LoRa Alliance standardized the LoRaWAN networking protocol [8] for LPWANs exploiting a chirp spread spectrum (CSS)-based technology developed and provided by the Semtech Corporation [9]. The third main potential solution offered on the market is Narrowband-IoT (NB-IoT), representing the result of a 3GPP (3rd Generation Partnership Project) standardization process begun in 2014 [10] and currently approaching its Release 15 [11]. The NB-IoT features originate from the LTE framework, with the employed frequency bands that are part of the LTE and GSM spectrum [12]. This is one of the marked differences with Sigfox and LoRa which, on the other hand, share the unlicensed ISM spectrum. However, each technology provides different performances [13].

Thanks to their characteristics, LPWANs have been recognized as particularly fitting for the IoT context, especially with respect to emerging scenarios such as remote health and industrial monitoring, smart cities and living. As shown in Table 1, different kinds of activities and measurements can be considered for each specific environment, therefore the use of technology that is as flexible and scalable as possible is fundamental to providing good network performance.

In medical applications, healthcare remote monitoring represents a promising solution to facilitate the interaction between patients and doctors. In fact, parameters such as blood pressure, temperature, or respiratory rate can be easily measured at home by patients through medical wearable devices, and then sent to a cloud server to make them available to hospital personnel at any time [14,15]. Monitoring activity also concerns industry, where production line control, inventory tracking and many other tasks can be performed in an automated and remote fashion. Finally, smart environment applications collect a wide range of activities to be handled, from traffic control, pollution monitoring and waste management in cities, to indoor lighting and climate control, energy and water use in buildings [16]. Overall, the diversity and specificity of each introduced application results in a largely variable exchanged data traffic volume. By looking, for instance, at the medical scenario, the data outputs of devices measuring blood pressure and heart rate are typically in the order of tens of bytes [17]. However, since these kinds of measurements are performed many times a day, it is possible to reach a daily aggregate data volume up to hundreds of bytes. On the other hand, processes such as electrocardiography (ECG) are more sporadic, but they can generate a large amount of information up to dozens of kilobytes [18]. Therefore, handling heterogeneous networks necessarily requests an efficient management of heterogeneous data.

The main aspect characterizing LPWANs is the restrained power consumption that brings benefits in terms of battery life extension, especially for mobile entities. On the other hand, the limited rate makes data transmission slower than in other technologies, so devices may be forced to remain connected for a long time. Energy saving strictly depends on how long devices stay active within the network, therefore this issue may become very challenging in those IoT applications that consider low interaction among entities. Specifically, the problem of power consumption in IoT LPWANs can be identified at the following levels:

- *network access and interference:* some technologies such as NB-IoT work according to a random-access procedure where a sequence of signaling messages is sent by the user asking for the channel resources.This mechanism may request non-negligible power since, if the channel is busy for a long time, the number of signaling messages to be generated will increase. However, the advantage of NB-IoT is that the use of licensed frequency bands limits the problem of interference with other communications. This is not true when dealing with systems such as Sigfox and LoRa where the channel access is simpler than the NB-IoT, but on the other hand exploiting the unlicensed spectrum for transmission may lead to strong interference, resulting in possible data packet loss. In that case, the information must be retransmitted, thus causing additional power usage.
- *exchanged data amount:* sometimes, when the communication is underway, some part of the data sent by the transmitting party may be already received at the receiving side due to not perfect synchronization. That information will be redundant and useless, and furthermore its transmission will lead to a waste of power. This occurrence is typical in remote file synchronization scenarios, where specific protocols are used to make the data transmission between two parties limited only to the new information. By doing so, the data traffic is reduced, optimizing the number packets to be exchanged and thus providing energy saving. Finally, the complexity requested by file processing and synchronization impacts on the device energy consumption, therefore the mechanisms for data communication must be not only efficient but also computationally feasible.

LoRa, Sigfox, NB-IoT and the other solutions used in LPWANs have different characteristics, therefore to manage the power consumption in the context of network access requires methods specifically tailored to the considered technology and application. On the other hand, data traffic optimization is independent from the network framework, so it would be possible to design some communication protocols providing good performance in the IoT environment in a more general way.

The main contribution of this work is therefore to consider well-known architectural models for IoT interconnection through LPWANs such as the ones discussed in [19] and provide a solution to reduce the data to be exchanged in the system for file synchronization purposes. A possible framework that this approach can refer to is e-health where on one side reliable file synchronization is needed, and on the other side both the energy consumption and the network load have to be reduced ([20,21]). Taking into account those concerns, we propose an adaptive file synchronization algorithm, particularly tailored to IoT applications, allowing:

- Data traffic optimization, to avoid the network overload;
- Energy saving, since reducing the amount of data to be transmitted allows the IoT device to limit power consumption.

It is worth noting that the proposal here identified can be applied independently to every low-power technology.

The rest of the paper is organized as follows. Section 2 reports the state of the art regarding file synchronization algorithms. In Section 3 the cloud-based network model under investigation is introduced, reporting the framework for the file synchronization procedure. Section 4 describes the proposed adaptive file synchronization algorithm, while Section 5 concerns the IoT devices power consumption analysis. Simulation results are shown and discussed in Section 6. Finally, Section 7 draws conclusions.

## 2. Related Works

The ever-growing network traffic and the size diversity of data to be exchanged have made the problem of remote file synchronization an ongoing issue to be tackled. Bandwidth saving, network latency minimization and overhead reduction are paramount, especially when the files to be updated usually exhibit only a few changes with respect to their previous version. In this scenario, the challenge is to perform the synchronization by identifying the similarities between two versions of the same file and hence by transmitting only the essential parts to the update.

In this context, one of the best-known synchronization algorithms [22] is *rsync* [23], originally developed for computer systems and still used by several applications. Considering a client-server scenario where the server has to be updated with the client file version, *rsync* operates the synchronization between the two parties by first splitting the server file into blocks (named chunks), each one identified through a double hash. The hash function is here employed only to compress a *d*-bytes chunk into a smaller *h*-bytes string. Moreover, the hash acts as a sort of signature for the corresponding chunk to be used for making chunk matching processing faster. Specifically, *rsync* considers each chunk as identified through a couple of hashes, referred as *signatures* in the rest of the paper. Then the list of these signatures is sent to the client and used to find matching blocks. Finally, the client generates a delta file, containing both the indexes of matching blocks and the literal bytes recognized as new, which is transmitted to the server for its file updating. *rsync* is therefore a single-round synchronization algorithm based on bidirectional communication between the two parties. This kind of framework has been considered in many other works such as in [24] where the features of *rsync* and a set of reconciliation techniques [25] are combined to achieve bandwidth saving. Overall, single-round-based techniques performance, including *rsync*, rely on the choice of a suitable block size. Sometimes, the number of transmitted bytes may be larger than the strictly necessary ones, leading to a performance reduction. Other strategies based on the *edit distance* are more efficient, but at the expense of a higher computational cost [26].

Several techniques following a multiple-rounds approach have been presented in the literature as well. An example is given in [27] where a two-phase synchronization protocol is proposed. The first step is represented by the so-called *map construction* where recursive block splitting is used to identify the common elements to both the parties. The second step instead concerns delta compression, which is the transmission of the unknown parts of the file necessary for the update. Despite multiple-rounds techniques providing significant improvements in terms of bandwidth efficiency, they may result in complex protocols, introducing non-negligible communication latencies and large overheads.

The paradigm of IoT has posed significant challenges that are different from those faced in *classic* distributed systems, therefore an important critical question recently arising in the context of IoT devices interaction and file synchronization is about the flexibility of the available algorithms in the IoT environment. To solve the potential efficiency reduction, several studies have been developed in the field of synchronization protocol optimization for IoT systems. Following this direction, the authors in [28] introduce a data synchronization technique between gateway and IoT platform that uses timestamp and bitmap to reduce byte traffic and latency.

Furthermore, handling the heterogeneity of the entities connected in the IoT is another aspect that may affect the performance of synchronization algorithms. The problem of data synchronization in multi-sensor scenarios is tackled in [29]. Specifically, a novel technique that uses the interactions and events experienced by each sensor within the network to solve multiple couplings among devices and efficiently handling the data stream synchronization is proposed.

Data exchange, storage, and sharing are instead specifically addressed for IoT LPWANs in [30], together with the other issues related to network management.

## 3. Remote File Synchronization in IoT LPWANs

### 3.1. Motivation and Goal

In the previous section we discussed how the remote file synchronization was mainly designed and employed in distributed systems (computer-based). On the contrary, only a few works have addressed this issue specifically for the IoT context and low-power communications.

We then analyzed the protocols that are efficiently conceived for low-power consumption, sporadic interaction among devices and reduced data traffic, and we identified the fundamental aspects to be taken into consideration in the design. Multiple-round synchronization techniques may not be efficient in a low-power scenario because they require devices to be active for a rather long time period, also increasing the amount of bidirectional information exchanged between the parties. On the other hand single-round approaches, such as the early *rsync* algorithm, [23] allow the communication to be simpler but the data traffic optimization could not always achieve maximum efficiency due to the limited adaptability of the protocols.

Aiming to address these issues, we propose a remote file synchronization algorithm that uses double signatures to perform file scanning and update just as *rsync* does but following an adaptive approach. In particular, the presented solution provides the dynamic adaptation of signatures and chunk size considered for the matching procedure. By doing so, the efficiency of single-round synchronization is improved, optimizing the data traffic as well.

We can describe briefly our proposal for remote file synchronization. Based on the same procedure used in *rsync* to find matching information between the file at the two parties, we introduce the following novel features the improve the performance of file synchronization:

- The adaptive tuning of the chunk dimension based on the distribution and type of modifications presented by the latest file version with respect to the previous one. By doing so, the file processing procedure and the amount of generated traffic are optimized.
- The dynamic selection between a signature-based and signature-free synchronization procedure, driven by the adaptation of the algorithm parameters, to exploit, when possible, the data compression provided by the signatures or, otherwise, avoid the use of signatures when it is not convenient due to the computational cost.

Finally, it is worth highlighting that, in general, file synchronization algorithms are implemented at the application layer of the OSI model, where it is safe to assume error-free and safe data communications. Data encoding, encryption, framing, and related issues are handled by other protocols at lower layers, therefore the addressing of these aspects goes beyond the scope of this work.

### 3.2. Reference Scenario

**Figure 1:**
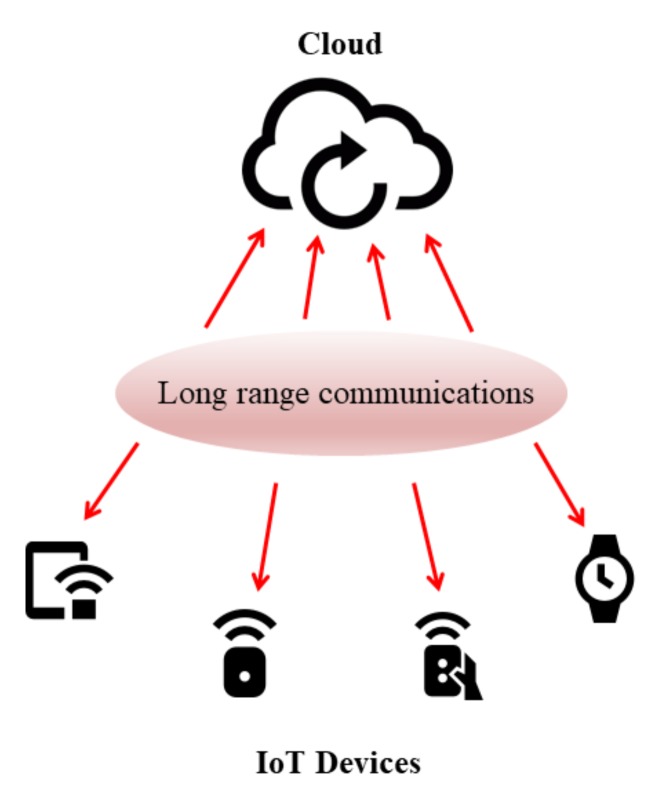
Typical IoT scenario where multiple devices are directly connected to the Cloud in a LPWAN.

Let us refer to an IoT scenario where a cloud server stores the updated data sent by multiple devices (smartphones, sensors, wearables) connected to the network [30]. Direct connection between cloud and IoT devices (DIoT) is assumed, considering a long-range communication infrastructure such as the one typically used in LPWANs (Figure 1). Therefore, the single DIoT–cloud interaction is depicted as follows.

**Figure 2:**
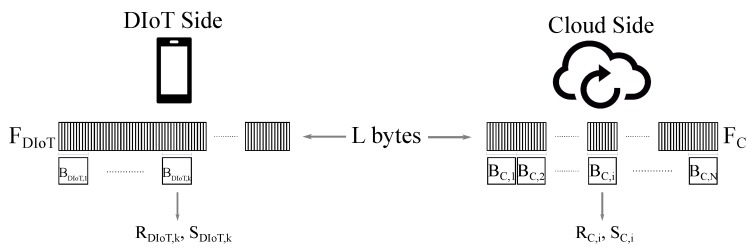
*rsync* procedure between DIoT and Cloud.

Let us consider two files $F_{C}$ and $F_{DIoT}$ at cloud and DIoT side, respectively (Figure 2). $F_{DIoT}$ is newer than $F_{C}$, therefore the cloud must receive from the DIoT the information to update $F_{C}$ to the latest version, that is $F_{DIoT}$. Without loss of generality, equal size is assumed for $F_{C}$ and $F_{DIoT}$. The steps performed for file synchronization are derived from the *rsync* algorithm, the essentials of which are reported below (see [23,31] for details).

First, the cloud organizes $F_{C}$ in non-overlapping blocks, named in the following as *chunks*, of size *d* bytes. Given the file dimension *L*, N=L/d chunks are obtained. For each chunk $B_{C,i}$ (with i=1,2,…,N) two checksums $R_{C,i}$ and $S_{C,i}$, a weak one and a strong one respectively, are calculated and sent to the DIoT side. Checksums are used to compress the chunks information so that the bytes to be transmitted are reduced.

The received sets $R=\{R_{C,1},R_{C,2},\ldots ,R_{C,N}\}$ and $S=\{S_{C,1},R_{S,2},\ldots ,R_{S,N}\}$ are used by the DIoT as a reference to process $F_{DIoT}$ and find potential matchings with $F_{C}$. Specifically, a moving window $B_{DIoT,k}$ of dimension equal to *d* bytes (*k* refers to the window offset along $F_{DIoT}$, with k=1,2,…,L) is used to scan $F_{DIoT}$. The DIoT calculates the checksums $R_{DIoT,k}$ and $S_{DIoT,k}$ of the current chunk $B_{DIoT,k}$ and searches for a matching with any element in R and S (the weak checksums are analyzed first, then, if there is a positive feedback, the strong checksums are compared to have the proof of chunks matching). If $B_{DIoT,k}$ is recognized to be already present in $F_{C}$, the DIoT saves the chunk index, referred as *token*. Otherwise, if no matching is found, the *k*-th byte of $F_{DIoT}$ is evaluated as *new* and therefore marked to be necessarily sent to the cloud. As the scanning of $F_{DIoT}$ goes on, the DIoT creates a delta file Δ containing tokens and literal bytes that will be ultimately transmitted to the cloud. Once Δ is received, the cloud performs the update of $F_{C}$ and recomputes the checksums on the new file chunks that will be used for the next synchronization events. Since the generated Δ reports the differences between $F_{DIoT}$ and $F_{C}$ files, the *rsync* algorithm is also named *rdiff*.

The good performance of *rsync* relies on the hypothesis that part of the file to be updated is sufficiently smaller than the entire file dimension. Furthermore, the size of chunks *d* and checksums *R*, *S* is set as fixed. As reported in [23], blocks dimensions between 500 to 1000 bytes are optimal. Regarding checksums, two 32-bit and 128-bit signatures are employed respectively to represent each chunk, therefore allowing a significant compression (32 + 128 = 160 bits, that is 20 bytes, are transmitted in place of a chunk of size equal to 500 bytes or more).

Computer systems for which *rsync* was initially designed usually consider files of sufficiently large size (tens of megabytes and more), therefore the introduced parameter configuration is appropriate. However, this fact may not be verified in IoT scenarios where limited amounts of data are managed. For instance, information about temperature and humidity in environmental monitoring systems or blood pressure measure in medical applications are represented by only a few bytes (at most kilobytes when considering aggregate measures).

In general, regarding the mechanism described in Figure 2, when the chunk dimension approaches the entire file size, finding matchings becomes ever more sporadic and the number of literal bytes to be transmitted grows. Consequently, the size of the file Δ grows, also increasing the data traffic. On the other hand, if the chunk size is reduced the compression ratio offered by the checksums decreases, therefore the advantage of using signatures is less significant.

## 4. Adaptive File Synchronization

The previous observations highlight the importance of a suitable parameter setup when dealing with file synchronization. However, in the literature, this issue seems to be handled in a somewhat general fashion, without properly considering how some aspects, such as the file update percentage, may significantly impact the protocol performance. Unfortunately, this kind of approach turns out to be inefficient in the IoT context with different connected entities exchanging different types of data, giving rise to heterogeneous and hardly predictable data traffic.

For these reasons, we propose an adaptive *rsync*-based file synchronization algorithm where the chunk size is dynamically tuned based on update distribution within the file, to optimize the number of literal bytes and tokens to be transmitted. Furthermore, the chunk dimension drives the choice between a signature-based or signature-free approach to be used.

**Figure 3:**
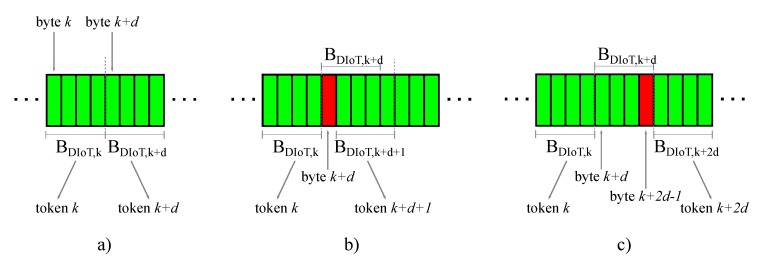
Examples of occurrences during file synchronization.

**Figure 4:**
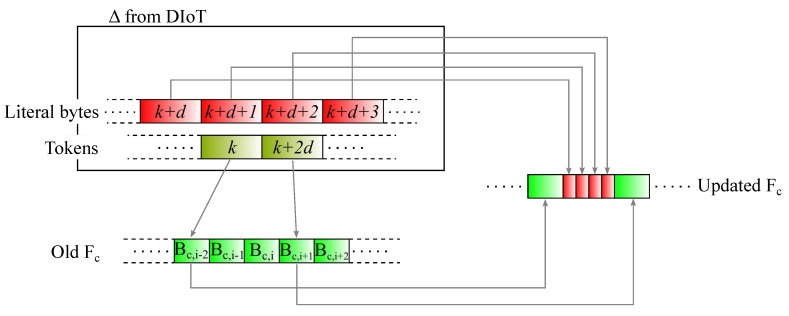
Cloud file update according to the scenario in Figure 3c.

Before detailing the algorithm, we summarize the following remarks about the original *rsync* mechanism:

- By referring to Figure 2, the search for matching chunks in $F_{DIoT}$ is performed by using a *d*-bytes sliding window $B_{DIoT,k}$ identified through the index of its first byte *k* (in Figure 2 the window moves from left to right, that is from first byte to last). As long as no matchings are found, the scanning proceeds by shifting the sliding window by a single position (*k* is incremented by a one unit). On the other hand, if the current chunk matches, the sliding window is moved by *d* positions (*k* is incremented by *d*).
- If the chunk $B_{DIoT,k}$ matches, a token is generated. Specifically, a token is an index reporting the position of the matched chunk within the file, therefore it will be represented exactly by the corresponding index *k*. An example of this occurrence is reported in Figure 3a, representing a portion of the scanned file $F_{DIoT}$. The sub-chunks in green refer to the bytes that are not changed with respect to the previous version of $F_{DIoT}$. In that case, the chunk $B_{DIoT,k}$ matches, therefore a token with index *k* is created. Then the file scanning continues considering the window $B_{DIoT,k+d}$. That chunk is matching too, leading to the generation of another token, identified by k+d.
- The generic chunk $B_{DIoT,k}$ is not matching if containing *at least* one single byte that is new with respect to the previous file version. This occurrence is described in Figure 3b–c, where the new bytes are marked in red.
  Specifically, in Figure 3a the chunk $B_{DIoT,k}$ is matching, but the following $B_{DIoT,k+d}$ is not because the (k+d)-th byte is a new one. Consequently, the (k+d)-th byte is added to the list of literal bytes to be sent to the cloud for update, and the sliding window is shifted by a single position. The next chunk $B_{DIoT,k+d+1}$ is recognized as known, therefore the relating token with index k+d+1 is sent.
  Regarding the case of Figure 3b, the new byte is *placed* at the end of $B_{DIoT,k+d}$, hence 4 single shifts are necessary before finding another matching chunk $B_{DIoT,k+2d}$. Interestingly, considering the generic chunk $B_{DIoT,j}$, as the file scanning moves on from the left to the right (that is, *j* increases from k+d to k+2d), the new byte slides from the right to the left within $B_{DIoT,j}$ until it completely moves out when j=k+2d. By doing so, the bytes from the (k+d)-th to the (k+2d−1)-th one are *all* judged as new and sent to the cloud. Actually, it would not have been necessary to transmit the bytes from the (k+d)-th to the (k+2d−2)-th one since they are not new, but in that case the size of the chunk *d* is too large, unavoidably leading to the transmission of unnecessary, redundant literal bytes.
  Let us note that the examples in Figure 3a,b consider only a single byte to be updated; however, the same discussion remains valid even if multiple new bytes are present in the file.
- At the end of the file analysis procedure, the generated Δ is sent to the cloud. Finally, synchronization is concluded by rearranging the cloud matching chunks (identified through the received tokens) and the literal bytes to obtain the updated version of $F_{C}$, as shown in Figure 4. The cloud will then compute the signature lists R and S referring to its latest file version, to be used when a new file synchronization procedure occurs.

### 4.1. Adaptive Chunking Algorithm

A fixed chunk-based synchronization mechanism does not allow sole and exclusive identification of the bytes being updated, as for the case of Figure 3b where unnecessary literal bytes are sent, thus increasing the final Δ size. However, by analyzing the indexes corresponding to the tokens (unchanged data) and literal bytes (new information) collected in Δ, it is possible to indicatively infer the amount and position of the updates within the file. This kind of information is then exploited for the adaptation of the chunk size *d* so to minimize the Δ dimension for the next file synchronization event. Specifically, we consider the file synchronization at time *t* resulting in a file $\Delta_{t}$ generated by the DIoT. As described in the previous sections, $\Delta_{t}$ is composed of both tokens and literal bytes. The basic approach of the proposed adaptive chunking is the following:

1. Having consecutive, adjacent, chunks (Figure 3a) suggests that the chunk size *d* could be increased in order to reduce the number of tokens (in the limit case, two *d*-bytes chunks corresponding to two tokens can be replaced by a unique 2*d*-bytes chunk expressed by a single token).
2. If the two consecutive matching chunks are not adjacent, it means that some updates are in between (Figure 3c). Therefore, *d* should be conveniently decreased to optimize the transmission of literal bytes, that is to reduce the redundancy.

The here-described possibilities must be evaluated on $P_{t}=\left\{p_{1},p_{2},\ldots ,p_{m},\ldots ,p_{M}\right\}$, defined as the set of tokens in $\Delta_{t}$ sorted in ascending order. Finally, the sum of the occurrences, suitably weighted, returns the new chunk size to be used for the next file synchronization event. The analysis of the sequence $P_{t}$ is conducted by considering two consecutive tokens at a time, namely $p_{m}$ and $p_{m-1}$, finally resulting in M−1 couples.

Given $d_{t}$ as the chunk size used in the current file synchronization event, we consider the difference $\Phi_{q}=p_{m}-p_{m-1}$, with q=m−1, hence q=1,2,…,(m−1),…,(M−1). As inferred by Figure 3a, having $\Phi_{q}=d_{t}$ reveals that the matching chunks $p_{m-1}$ and $p_{m}$ are adjacent, therefore leading to the conclusion at point-1 introduced above. Furthermore, if the measure of Φ remains constant and equal to $d_{t}$ as *q* grows, then it means that there are multiple adjacent chunks identifying a portion of the file that does not need to be modified. The number of adjacent chunks $N_{ac}$ defines the size of the unmodified portion and it is used to drive the adaptive chunking as follows:

(1) $$d_{t+1,w}=d_{t}+\mu N_{ac}$$

According to Equation (1), the chunk dimension is increased as a function of the number of detected adjacent chunks composing the *w*-th file section. The parameter μ acts as a step size ruling the speed of adaptation.

On the other hand, measuring $d_{t}<\Phi_{q}\leq 2d_{t}$ (Figure 3b,c) shows that there is at least an updated byte between the chunks $p_{m-1}$ and $p_{m}$, resulting in point-2 of the considered possibilities. This kind of approach does not provide the exact knowledge of both the number and position of the new bytes; however, we remark that the first step of the proposed algorithm concerns only an approximate description of the update distribution along the file $F_{DIoT}$. Therefore, in this direction, it is sufficient for us to detect the minimum number of updated bytes. Specifically, given $p_{m}$ and $p_{m-1}$ so that $ud_{t}<\Phi_{q}\leq \left(u+1\right)d_{t}$, with $u=1,2,\ldots ,\left\lfloor \frac{L-m}{d_{t}}\right\rfloor$, we measure the minimum number of updated bytes $N_{UD}$ as:

(2) $$N_{UD}=\left\lceil \frac{\Phi_{t}}{d_{t}}-1\right\rceil$$

Therefore, in this case, the adaptation rule for chunk size decrease is given by:

(3) $$d_{t+1,w}=d_{t}-\mu N_{UD}$$

Equations (1)–(3) return a partial metric of the new chunk size that refers only to the considered file section *w*. Depending on the update distributions along the file $F_{DIoT}$, the proposed algorithm provides *W* partial measures corresponding to the *W* identified file portions, so that the final resulting averaged chunk size $d_{t+1}$, to be used in the next (t+1)-th synchronization procedure, is obtained by:

(4) $$d_{t+1}=\frac{1}{W}\sum_{w=1}^{W}d_{t+1,w}$$

As introduced before, the step size μ defines the adaptation speed of the algorithm. An opportune choice of μ should be driven by the *temporal* features of the file $F_{DIoT}$. In fact, for applications where the update distribution along $F_{DIoT}$ changes quite slowly in time (that is, considering the synchronization events at time *t* and t+1, the updated bytes in $F_{DIoT,t}$ and $F_{DIoT,t+1}$ are localized in the same portion of the file) it would be convenient to select a high-valued μ, making the new chunk size the optimal one as quickly as possible. A similar behavior can be found in medical device networks where the output files to be exchanged are usually formed by a header, reporting barely variable control information, and a payload containing the actual measurement. On the other hand, if there is no correlation between the consecutive update distribution in $F_{DIoT,t}$ and $F_{DIoT,t+1}$, working with a relatively small μ is preferable since by doing so the chunk dimension varies slowly, therefore avoiding large deviations from the optimal value. This happens especially when the rough data contained within the file are larger than the data header.

### 4.2. On Double Signature Efficiency

The possibility of performing an adaptive chunking shows the importance and efficiency of double signature, to be discussed. In *rsync*-based file synchronization strategies, the size of chunks and signatures employed for data compression is fixed and no parameter variations are expected. As already mentioned in Section 3.2, *rsync* considers the use of 20-byte signatures ($d_{R}$ = 4 bytes for the rolling checksum, $d_{S}$ = 16 bytes for the strong checksum) to compress chunks of dimension typically greater or equal to 500 bytes [23], resulting in a compression ratio $r_{c}=\frac{d}{d_{R}+d_{S}}\geq 15$. This parameter setup is particularly suited to the context where the files to be synchronized are sufficiently large and the percentage of updates is quite low; on the other hand it may be failing when dealing with small-size data subject to significant changes in time. The proposed adaptive chunking algorithm solves part of the problem by dynamically changing the size of the chunk according to the update distribution, but it does not care about signature efficiency. Specifically, when several synchronization procedures consider a large number of changes on the same file, the adaptive chunking algorithm returns a decreasing chunk size *d*. In this context, having $d=d_{R}+d_{S}$ represents the signatures performance lower bound, that is where there is no compression gain. Actually, the use of signatures becomes even totally disadvantageous when $d<d_{R}+d_{S}$ (the chunk is extended instead of compressed).

Therefore, to overcome the potential failures of double signature, we present two different solutions for the choice of the most opportune signatures dimension that are driven by the chunk size returned by the previously described algorithm (the essentials are reported in Algorithm 1).

- The first strategy considers a lower bound on the chunk dimensioning, that is, given the rolling and strong checksums dimension $d_{R,ref}$ and $d_{S,ref}$ respectively, the following condition must be met:
  (5) $$d_{t+1}>\theta_{c}=d_{R,ref}+d_{S,ref}$$
  with $\theta_{c}$ being the reference threshold. Hence Equation (5) represents a sort of additional step to the adaptive chunking algorithm in Section 4.1 that prevents the estimated new chunk size to be smaller than the signatures total dimension. By doing so the benefits of double signature are preserved, but the potential provided by the adaptive chunking algorithm is not fully exploited. Based on the *rsync* framework we have considered, the proposed chunking algorithm and Equation (5), we refer to the overall file synchronization procedure as Adaptive Chunking rdiff (AC-*rdiff*).
- The second proposed approach relies on a threshold-based mechanism offering the possibility to dynamically move from a signature-based procedure to a signatures-free one and vice versa. In particular, the same threshold $\theta_{c}$ defined as in AC-*rdiff* is considered to evaluate the convenience of using signatures as follows:
  (6) $$\mathrm{Synchronization}=\left\{\begin{array}{c} \mathrm{Hashing}-\mathrm{free}\;\mathrm{if}\;d\leq \theta_{c}\;\to d_{R,t+1}=0;d_{S,t+1}=0\\ \mathrm{Hashing}-\mathrm{based}\;\mathrm{if}\;d>\theta_{c}\;\to d_{R,t+1}=d_{R,ref};d_{S,t+1}=d_{S,ref} \end{array}\right.$$
  If the chunk size is over the threshold $\theta_{c}$, the signatures-based approach is selected which can bring benefits in terms of data compression. On the other hand, when the chunk dimension is under the reference threshold, the file-scanning procedure for matching detection is performed directly on the *original* chunks without resorting to any compression. For representation purposes, in Equation (6) and in Algorithms 2 and 3 the dimensions of signatures to be employed in the next t+1 synchronization procedure, namely $d_{R,t+1}$ and $d_{S,t+1}$, are set to $d_{R,ref}$ and $d_{S,ref}$ for the signature-based approach, and to 0 for the signature-free synchronization. Because of the provided signatures on-off switching feature, we define this solution as Adaptive Hashing rdiff (AH-*rdiff*). In contrast to AC-*rdiff* where the chunk size choice is dependent of (but also constrained to, when referring to the minimum allowed size) the signature dimension, with the AH-*rdiff* algorithm the use of signatures is defined by the chunk size.

**Algorithm 1** Procedure for adapting the chunk size
  1:**Input:** Current chunk size $d_{t}$; Token index vector $P_{t}$  2:*$W_{sum}$ = 0* chunk estimate sum metric  3:*w = 0* step counter  4:*$N_{ac}$ = 0* No. consecutive, unmodified chunks  5:
**for** *m = 2:M*
  6:    i=m−1  7:    $\Phi_{i}=p_{m}-p_{m-1}$ Token difference  8:    **if** $\Phi_{i}==d_{t}$  9:     **$N_{ac}=N_{ac}+1$**10:    **else**11:       **if** *$N_{ac}>0$*12:         *w = w + 1*13:         $d_{t+1,w}=d_{t}+\mu N_{ac}$14:         *$W_{sum}$ = $W_{sum}$ + $d_{t+1,w}$*15:         $N_{ac}=0$16:       **end if**17:       $N_{UD}=\left\lceil \frac{\Phi_{t}}{d_{t}}-1\right\rceil$ No. updates detected18:       *w = w + 1*19:       $d_{t+1,w}=d_{t}-\mu N_{UD}$ Partial chunk estimate20:       *$W_{sum}$ = $W_{sum}$ + $d_{t+1,w}$*21:    **end if**22:
**end for**
23:$d_{t+1}=W_{sum}/w$ Final chunk size estimate24:
**return** $d_{t+1}$



**Algorithm 2** AC-*rdiff*
1:**Input:** chunk size estimate $d_{t+1}$; Reference signatures size $d_{R,ref},d_{S,ref}$2:$\theta_{c}=d_{R,ref}+d_{S,ref}$ Threshold3:
**if** $d_{t+1}\leq \theta_{c}$
4:  $d_{t+1}=d_{R}+d_{S}+1$5:
**end if**
6:
**return** $d_{t+1}$



**Algorithm 3** AH-*rdiff*
  1:**Input:** Chunk size estimate $d_{t+1}$; Reference signatures size $d_{R,ref},d_{S,ref}$  2:$\theta_{c}=d_{R,ref}+d_{S,ref}$ Threshold  3:
**if** $d_{t+1}\leq \theta_{c}$
  4:  $d_{R,t+1}=0$  5:  $d_{S,t+1}=0$  6:
**else**
  7:  $d_{R,t+1}=d_{R,ref}$  8:  $d_{S,t+1}=d_{S,ref}$  9:
**end if**
10:**return**$d_{R,t+1}$, $d_{S,t+1}$


### 4.3. Output Delta Format and Signatures Reference Lists Update

**Figure 5:**
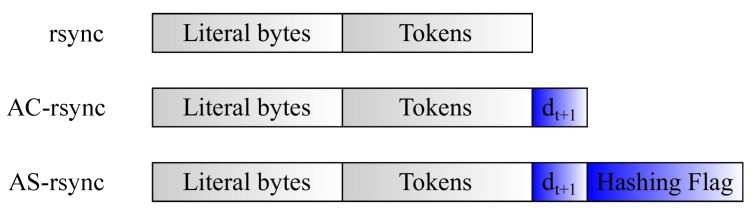
Delta structure in original *rsync*, AC-*rdiff* and AH-*rdiff* respectively.

At the end of the file synchronization occurring at time *t*, the AC-*rdiff* and AH-*rdiff* algorithms return a new adapted chunk dimension to be considered during the next file synchronization procedure at time *t* + 1. Concerning AH-*rdiff*, the choice between the signature-based and signature-free synchronization approach is also determined. This reference information must be known also from the cloud, so that it can adequately handle its file update and signature generation in the next synchronization procedure. Therefore, the delta output file $\Delta_{t}$ sent by the DIoT will contain not only the literal bytes and tokens to be used for file update, but also another field reporting the new chunk size $d_{t+1}$. Furthermore, in AH-*rdiff*, an additional flag is employed for signaling if the use of signatures is convenient or not (Figure 5). However, it is worth noting that the addition of $d_{t+1}$ and flag has in general an absolutely negligible impact on the delta file dimension since they can be represented in principle by only a few bits (at most, a couple of bytes).

As detailed in Section 3.2, the original *rsync* algorithm entails that every time a synchronization procedure is performed, the cloud generates two lists of reference signatures, R and S and sends them to the DIoT in order to start a new synchronization procedure. However, the presence of this download transmission may not be convenient, especially in networks where costs are dependent on the amount of data traffic exchanged. For instance, service fares proposed by some NB-IoT network providers are bounded to a fixed traffic threshold, beyond which additional costs may be required [32,33]. Therefore, while the uplink data traffic is optimized thanks to the use of AC-*rdiff* and AH-*rdiff*, a proper management of downlink communication should be also provided to save spectral resources and limit the potential costs related to the traffic volume.

In this direction, we present a solution where the DIoT generates its own reference signature lists, so that the downlink interaction with the cloud is limited to very sporadic cases. Specifically, the idea is that once the adaptive algorithm (AC-*rdiff* or AH-*rdiff*) has returned the new chunk size $d_{t+1}$, the DIoT organizes its current file $F_{DIoT,t}$ in chunks of size $d_{t+1}$ bytes and calculates the corresponding signatures sets R and S to be used as reference in the next file synchronization. It is worth highlighting that this procedure is exactly the same as performed by the cloud in the original *rsync* framework. In that case, the cloud generates the new signatures lists on its updated file $F_{C,t}$, entailing that $F_{C,t}$ has become equal to $F_{DIoT,t}$. Therefore, the sets R and S calculated by the cloud on $F_{C,t}$ will be the same ones computable from $F_{DIoT,t}$ at DIoT side. The proposed reference signatures self-generation approach avoids the cloud to spend data traffic to transmit R and S, but it also provides energy saving as explained in the next section. On the other hand, it requires a further computational effort from the DIoT that can however be considered as negligible since the device’s power consumption mainly concerns data transmission and reception mechanisms. Finally, it is worth noting that the downlink communication from the cloud to the DIoT is significantly reduced but not completely cut. In fact, the signature lists transmission from the cloud remains available and acts as a sort of reset when unexpected events, such as DIoT formatting, malfunctions, or failed updates, occur, compromising the DIoT-cloud connection.

## 5. Power Consumption Analysis

In general, power consumption is not considered a critical issue for the cloud since it is referred to as an actively powered entity (as a data center is). On the other hand, energy saving is fundamental for IoT devices that are typically battery supplied, to support mobility. Following the model given in [34], the total energy consumption for IoT devices can be derived as the sum of four components:

(7) $$E_{tot}=E_{tx}+E_{rx}+E_{prc}+E_{sys}$$

where $E_{tx}$ refers to data communication, $E_{rx}$ to sensing and data reception, $E_{prc}$ to the processing and $E_{sys}$ to the other minor functionalities of the considered device. However, by assuming $E_{sys}$ is negligible and considering $E_{prc}$ as included in data transmission and reception processes, it is possible to recast Equation (7) in the classic energy consumption models developed for Wireless Sensor Networks (WSNs):

(8) $$E_{tot}=E_{tx}+E_{rx}=P_{tx}\frac{D_{s}}{v_{UL}}+P_{rx}\frac{D_{r}}{v_{DL}}$$

with the total energy consumption of DIoT being described as a function of their transmission and reception activity [35,36]. Specifically, the transmit power $P_{tx}$ takes into account the energy spent to run the electronic hardware (mainly related to the digital-to-analog signal conversion and power amplification), while the received power $P_{rx}$ is defined by the energy employed for maintaining the device in active mode and processing the received signals [37]. $v_{DL}$ and $v_{UL}$ refer to the download and upload data rate, respectively. Finally, $D_{s}$ and $D_{r}$ represent the data volume to send and receive. In particular, considering Equation (8) in the context of data synchronization returns $D_{s}$ as the Δ containing the information transmitted from the DIoT to the cloud for its update, and $D_{r}$ as the reference information (that is the signatures lists) sent by the cloud to let the DIoT perform its file processing and Δ generation. The parameters $P_{tx}$, $P_{rx}$, $v_{DL}$ and $v_{UL}$ are instead specific to the employed technology, therefore not dependent of the data synchronization mechanism. As an additional comment, it is worth noting that the implementation of AC-*rdiff* and AH-*rdiff* algorithms in a communication framework with reduced cloud-to-DIoT transmission such as one proposed in the previous section returns the following benefits. First, the optimization of Δ, that is $D_{s}$, implies the optimization of $E_{tx}$. Second, the reference signatures self-generation approach allows a significant reduction of $D_{r}$, therefore making the $E_{rx}$ negligible with respect to $E_{tx}$. Therefore, we show that $E_{tot}$ becomes mainly dependent of $E_{tx}$.

Battery-supplied DIoTs are typically designed for sporadic data transmissions, thus remaining idle for most of time, except for narrow active mode time windows. Time and energy spent in both these possible states allow the device battery lifetime to be measured as [38]:

(9) $$L\left(t_{e}\right)=\frac{C\cdot SF}{E_{tx}\left(t_{e}\right)+E_{bg}}$$

where *C* and SF is the battery capacity and safety factor, respectively, $E_{tx}$ the average energy consumption component taken from Equation (8) and $E_{bg}$ the DIoT background electronic hardware consumption. Both $E_{tx}$ and *L* are averaged on $t_{e}$, representing the time interval between two consecutive data transmissions (that is, two file synchronization events).

Finally, we would like to remark that the power consumption analysis here reported has been rephrased to consider those aspects characterizing the file synchronization at the application layer. Moving down towards the network access layer, cloud-DIoT handshaking mechanisms and data framing are performed. Moreover, each specific layer protocol considers the introduction of additional information. Therefore, as the weight of $D_{s}$ and $D_{r}$ might be different from the one considered for our purpose, the model in Equations (8) and (9) should be conveniently handled. However, since Δ represents the largest part of the total amount of data exchanged between two nodes, we believe that the proposed analysis may help to emphasize the importance of an efficient data synchronization protocol, especially in low-power IoT scenarios.

## 6. Numerical Results

The performance of the proposed AC-*rdiff* and AH-*rdiff* algorithms have been evaluated in terms of generated data traffic amount and DIoT power consumption. To this aim, we have developed a MATLAB-based simulator where the file synchronization between two parties, e.g., a DIoT and the Cloud, has been implemented. Specifically, first a byte string *A*, representing the file stored at cloud side, is created. Then another string, namely *B*, equal to *A* except for some modified parts, is generated. *B* acts as the file at DIoT side, so *A* is updated to *B* by resorting to different synchronization algorithms. Simulations have considered files, the dimension of which is equal to 3 kB. Taking into account the discussion reported in Section 1, the choice of such file dimension makes the simulation framework fairly realistic with respect to the scenarios described in Table 1 where limited-size data are handled (the file may represent some kind of measure coming from a sensor, or it may contain multiple information collected within a time interval but to be sent only at specific time hours of the day). Specifically, we have implemented file synchronization according to the following algorithms:

- ${\mathrm{rsync}}_{32,128}$: the classic *rsync* mechanism as in [23] that considers the use of a 32-bit rolling checksum and a 128-bit MD5 hash, with a static chunk size equal to 500 bytes.
- ${\mathrm{rdiff}}_{32,128}$: the algorithm parameters are the same used in [23], except for the chunk size that is set to 40 bytes, so letting the double signature provide a compression ratio $r_{c}=2$ (the name *rdiff* is used only to differentiate the current algorithm from the previous *rsync*).
- ${\mathrm{rdiff}}_{16,64}$: as the dimension of the files to be synchronized is limited, two smaller signatures are considered, a 16-bit rolling checksum and a 64-bit cyclic redundancy check respectively, with the chunk size equal to 20 so to obtain $r_{c}=2$.
- $\mathrm{AC}-{\mathrm{rdiff}}_{16,64}$: the proposed algorithm uses a 16-bit rolling checksum and a 64-bit cyclic redundancy check as signature, while the chunk size is dynamically adapted (the step size in Equations (1)–(3) was set to μ = 0.5).
- $\mathrm{AH}-{\mathrm{rdiff}}_{16,64}$: based on the chunk size returned by the adaptive algorithm (μ = 0.5), either a signature-based approach with a 16-bit and 64-bit signatures or a signature-free procedure is used.

All the algorithms listed above are implemented within a synchronization framework where the DIoT calculates by itself the reference signatures without receiving them from the cloud (an exception is represented by the very first synchronization event when the DIoT is initialized with the reference signatures coming from the cloud).

### 6.1. Data Traffic Performance

**Figure 6:**
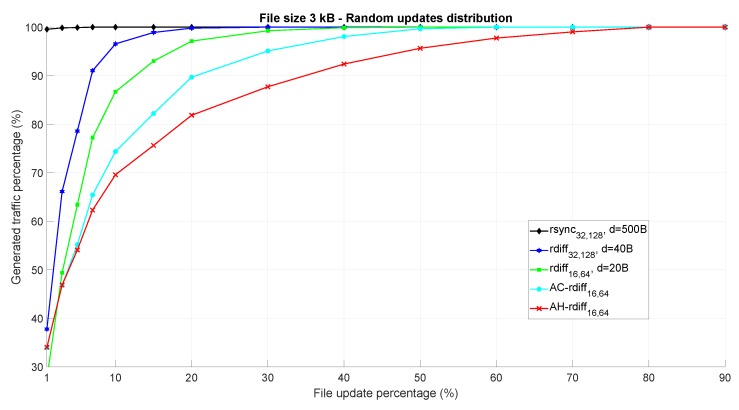
Performance of the considered file synchronization algorithms as a function of the file update percentage. Random update distribution within the file is assumed.

Figure 6 shows the amount of generated data traffic for the considered synchronization algorithms, expressed as a percentage with respect to the entire file dimension (having 100% of generated traffic means essentially that the DIoT is sending the whole file to the cloud). The changed bytes have been individually, randomly sorted within the file $F_{DIoT,t}$. The results, averaged over 100 simulations, are a function of the file update percentage, indicating how much the current file $F_{DIoT,t}$ is changed with respect to its previous version $F_{DIoT,t-1}$.

As expected, ${\mathrm{rsync}}_{32,128}$ is completely inefficient as the chunk size *d* = 500 bytes is too large with respect to the entire file dimension. In fact, it is very hard to find matching chunks, especially when the update percentage grows. On the other hand, ${\mathrm{rdiff}}_{32,128}$ and ${\mathrm{rdiff}}_{16,64}$ show better performance since the considered chunk size is much smaller than in 500 bytes. However, in both cases the generated data traffic reaches 100% rapidly as the update percentage of the file increases. Finally, $\mathrm{AC}-{\mathrm{rdiff}}_{16,64}$ and $\mathrm{AH}-{\mathrm{rdiff}}_{16,64}$ provide best performance thanks to the fact that the chunk size is dynamically tuned according to the measured update percentage. This fact can be appreciated especially for high update percentages, where other fixed chunk size-based algorithm are inefficient. As explained in Section 4.2, the AC-*rdiff* algorithm considers a lower bound regarding the minimum chunk size, while AH-*rdiff* has no constraints since if the chunk dimension is lower than the signature sum size, the system simply switches from the signature-based to the signature-free approach. For this reason, $\mathrm{AH}-{\mathrm{rdiff}}_{16,64}$ is better performing than $\mathrm{AC}-{\mathrm{rdiff}}_{16,64}$.

**Figure 7:**
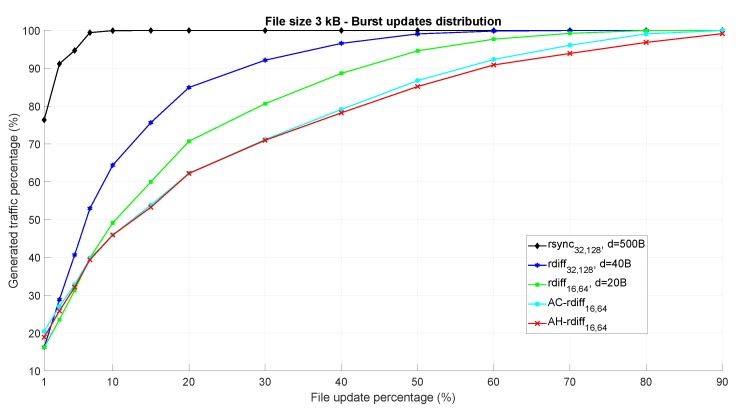
Performance of the considered file synchronization algorithms as a function of the file update percentage. Burst updates distribution within the file is assumed.

The same analysis reported in Figure 6 has been performed considering a different scenario where the updated bytes within $F_{DIoT}$ are characterized by a burst distribution, the dimension of which is sorted following a Gaussian probability density function with mean $\mu_{burst}$ = 5 and variance $\sigma_{burst}^{2}$ = 6.25 (the size of new byte chunks ranges from 2 to 15 bytes). From a data synchronization point of view, this latter case is more favorable than the previous one where the bytes to be changed were individually and randomly distributed since, for a given update percentage, having a more compact distribution of the updates returns a higher possibility to find matching chunks, thus reducing both the processing time and the amount of bytes to be transmitted to the cloud. This fact is confirmed by the results in Figure 7 where it is possible to observe how all the considered algorithms show better performance with respect to the previous case of Figure 6. However, the higher efficiency of AC-*rdiff* and AH-*rdiff* mechanisms is clearly evident even in this scenario.

**Figure 8:**
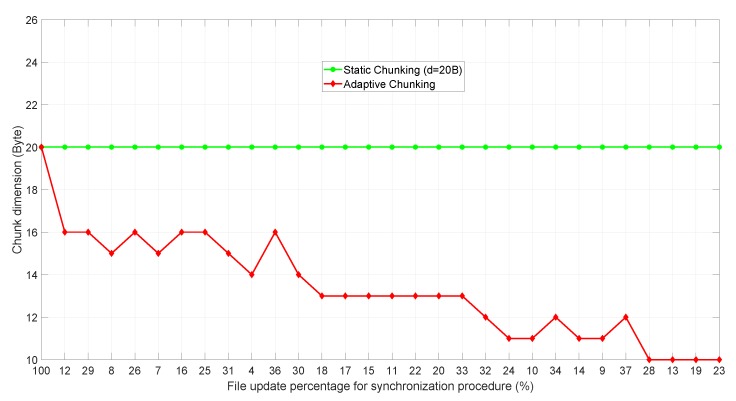
Chunk dimension adaptation as the synchronization events occur.

Finally, we have simulated 30 consecutive file synchronization procedures, reporting in Table 2 the average traffic percentage generated according to the algorithms under investigation. The first column of Table 2 also shows the standard deviation of the results, which is quite uniform for all the considered algorithms except for ${\mathrm{rsync}}_{32,128}$. This is because ${\mathrm{rsync}}_{32,128}$ generates an average data traffic that essentially approaches the entire file size of 3 kB, so the deviation is very small. On the other hand, with the other algorithms returning lower average results, the standard deviation assumes larger values. For each considered synchronization event, the file update percentage has been sorted following a log-normal distribution with $\mu_{ud}$ = 2.5 and $\sigma_{ud}$ = 0.8 (the update percentage associated with the *s*-th synchronization procedure, with *s* = 1, 2, …, 30, indicates how much the file at time *t* = *s* is changed with respect to its previous version at time *t* = s−1). The changed bytes within the file have been instead burst-sorted. Furthermore, Table 2 also shows the chunk dimension considered by each algorithm. For AC-*rdiff* and AH-*rdiff* the mean value is reported, which is identical since the chunk size is adapted following the same approach (left column of Table 2). Specifically, in Figure 8 it is possible to appreciate how the chunk dimension dynamically changes in time (the figure also reports the chunk dimension considered in ${\mathrm{rdiff}}_{16,64}$ a reference value). The *x*-axis reports 30 ticks corresponding to the 30 synchronization procedures, each one labeled with the respective considered file updated percentage value. The trend of the chunk size adaptation strictly depends on the file update percentage. In fact, for high percentage values the chunk size decreases, while for small updates the chunk dimension grows. When instead the chunk does not change, it means that despite different update percentages, the algorithm has recognized no convenient size variations.

As an additional comment, we observe that the choice of file dimension equal to 3 kB does not limit validity of the results, since the goal of the proposed analysis is to evaluate the byte traffic-saving percentage provided by the considered algorithms. In fact, file synchronization protocols can handle dimension mismatching between the DIoT and cloud files. In general, it is important to observe that, when the chunk dimension is similar to the entire file size, static chunk-based algorithms are inefficient since it is hard to find matching chunks if a significant part of the file needs to be updated. On the other hand, when the file dimension grows with respect to the chunk, the performance improves. However, thanks to the adaptive approach, AC-*rdiff* and AH-*rdiff* are essentially not sensitive to the file size, thus providing good performance in any scenario.

### 6.2. Power Consumption Performance

**Figure 9:**
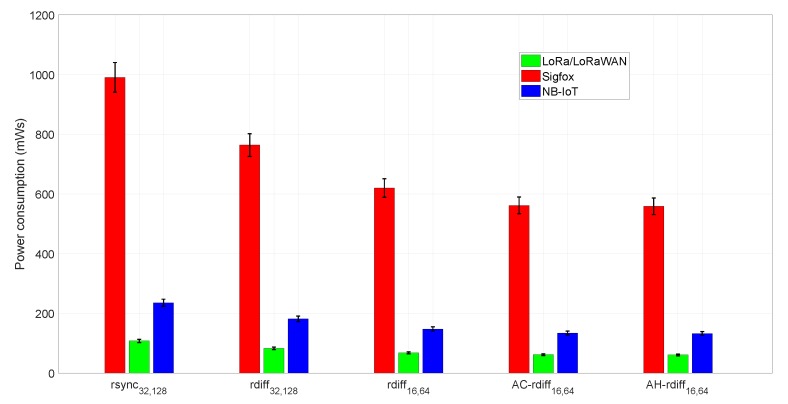
Power consumption, measured in milliwatt second, of a single synchronization procedure considering different LPWAN technologies and algorithms.

The results obtained from the data traffic analysis have been exploited to measure the DIoT power consumption. The reference signatures self-generation approach characterizing the DIoT allows the energy spent for data reception to be neglected, so we have simplified the measure of power consumption of Equation (8) by only referring to the transmission component. By doing so, we can evaluate how the byte traffic saving provided by the different synchronization algorithms impacts on the device power consumption and battery lifetime. Specifically, the average data traffic reported in Table 2 has been considered as the transmitted data volume $D_{s}$. Concerning the other parameters $P_{tx}$ and $v_{UL}$ introduced in Equation (8), we refer to the most popular LPWAN technologies on the market, namely Sigfox [7], LoRa/LoRaWAN [39,40], and NB-IoT [10] (Table 3). Therefore, we have evaluated the DIoT energy consumption of a single synchronization procedure for different algorithms as shown in Figure 9, with the error bars referring to a 95% confidence interval.

Finally, the measures reported in Figure 9 have been exploited to estimate the battery lifetime following Equation (9). Regarding the battery parameters, we have chosen *C* = 27.7 Wh and SF = 1/3 as in [38,41]. The background device power consumption has been neglected to make the analysis specifically referring to the data exchange context. Furthermore, we have considered the scenario where a file synchronization procedure is performed once a day, thus returning a $t_{e}$ = 24 h. Therefore, the battery lifetime, expressed in years, is described in Table 4 as a function of the considered LPWAN technologies and file synchronization algorithms.

By jointly observing the results in Figure 9 and Table 4 it is possible to appreciate how the data traffic saving provided by AC-*rdiff* and AH-*rdiff* returns, as expected, significant benefits in terms of DIoT energy consumption. Furthermore, it is also evident that the amount of data exchanged impacts on the performance of the employed LPWAN technology. In fact, traffic volumes in the order of kilobytes (as considered in the simulations) makes use of Sigfox unfeasible since the very low data rate characterizing this technology unavoidably leads to long transmission times and, therefore, to high power consumption. On the other hand, LoRa/LoRaWAN and NB-IoT are better suited to the considered traffic volume. However, the presented scenario with 3 kB files daily updating is quite unusual in the IoT, since the amount of exchanged data is typically much lower. We have intentionally chosen this framework to stress the performance provided by the proposed synchronization algorithms. Moreover, considering more realistic circumstances where the daily traffic volume is in the order of a few hundreds of bytes, the battery lifetime values in Table 4 will increase, reaching the 5–10 years life usually claimed for IoT devices.

## 7. Conclusions

This paper dealt with data synchronization in IoT LPWANs. In this context, two adaptive algorithms, namely AC-*rdiff* and AH-*rdiff*, have been presented to optimize the amount of data to be exchanged when the synchronization between a DIoT and the Cloud is performed. The aim is to reduce the amount of traffic exchanged, still preserving the ideal synchronization, thus saving energy and increasing the DIoT lifetime. The performance analysis here reported has highlighted a significant traffic volume saving when the proposed algorithms are used with respect to other solutions currently adopted in remote synchronization. Furthermore, reducing the data to be transmitted leads also to improve the DIoT energy saving, representing a very challenging issue in the IoT, and therefore extending device battery lifetime. Future works will consider a more complex scenario where multiple DIoTs share the same cloud storage resources. In that case, an opportune time synchronization protocol among parties must be provided to let both the cloud and DIoTs perform file updates without incurring problems of access and worthless data duplication.

## Figures and Tables

**Table 1:** Typical IoT LPWANs use cases.

IoT Scenario		Applications		Typical Traffic Volume
		Respiratory rate		
		ECG		Tens of bits to a few kilobytes
*Healthcare*		Blood pressure		per measure
		Skin temperature		(single/aggregate measures messages)
		Oxygenation		
		Environment monitoring		
*Industry*		Indoor localization		Tens of bits per message
		Production line control		
		Traffic management		
*Smart*		Waste management		Tens of bits per message
*cities*		Parking tracking		(aggregate data measures)
		Pollution monitoring		
		Lighting control		
*Smart buildings*		Energy/water use		Hundreds of bits per message
*and living*		Surveillance		(single/aggregate data measures)
		Indoor climate control		

**Table 2:** Average data traffic generated by different synchronization algorithms.

	Average Traffic (Standard Dev.)	Average Traffic Percentage	Chunk Dimension
	(kByte)	(%)	(Byte)
${\mathrm{rsync}}_{32,128}$	2.96 (0.03)	98.89	500
${\mathrm{rdiff}}_{32,128}$	2.28 (0.49)	76.31	40
${\mathrm{rdiff}}_{16,64}$	1.85 (0.47)	61.72	20
$\mathrm{AC}-{\mathrm{rdiff}}_{16,64}$	1.68 (0.41)	56.01	14
$\mathrm{AH}-{\mathrm{rdiff}}_{16,64}$	1.67 (0.39)	55.94	14

**Table 3:** LPWANs Technologies Parameters.

	$P_{\mathrm{tx}}$ (dBm)	$v_{\mathrm{UL}}$ (kb/s)
**Sigfox**	14	0.6
**LoRa/LoRaWAN**	14	5.5
**NB-IoT**	23	20

**Table 4:** Device battery lifetime, measured in years, considering the scenario where the 3 kB file synchronization is performed once every 24 h. Results are shown as a function of the LPWAN technology and file synchronization algorithm.

	Sigfox	LoRa/LoRaWAN	NB-IoT
${\mathrm{rsync}}_{32,128}$	0.10	0.85	0.39
${\mathrm{rdiff}}_{32,128}$	0.12	1.10	0.50
${\mathrm{rdiff}}_{16,64}$	0.15	1.34	0.62
$\mathrm{AC}-{\mathrm{rdiff}}_{16,64}$	0.17	1.47	0.68
$\mathrm{AH}-{\mathrm{rdiff}}_{16,64}$	0.17	1.50	0.69
